# Building pathology capacity in sub-Saharan Africa to improve breast cancer diagnosis and treatment: training laboratory technicians in high-quality manual immunohistochemistry

**DOI:** 10.1186/s12885-023-11756-8

**Published:** 2024-01-03

**Authors:** Linda Setiawan, Katy Graef, Dan Schmolze, Alemwosen Alem, Lesley Taylor

**Affiliations:** 1 BIO Ventures for Global Health, Seattle, WA USA; 2 Department of Pathology, City of Hope Comprehensive Cancer Center, Duarte, CA USA; 3 Pathology Department, Hawassa University College of Health Science, Hawassa, Ethiopia; 4 Department of Surgery, City of Hope Comprehensive Cancer Center, Duarte, CA USA

**Keywords:** Education intervention, Pathology, Breast cancer, Sub-Saharan Africa, Immunohistochemistry

## Abstract

**Background:**

To address the need for a skilled workforce in breast cancer (BC) pathology in sub-Saharan Africa (SSA), we implemented an education program to train laboratory technicians in manual immunohistochemistry (IHC).

**Methods:**

A quality improvement education project was developed. Interactive webinars were held every six months with didactics and presentations from African experts with experience in IHC. We conducted knowledge assessments and surveys on current practice, equipment, and human resources. A digital mentorship platform (DMP) was created for discussions, sharing SOPs, and networking. For one year (2022–2023), we followed developments in pathology capacity, practice changes, and educational needs. A paired t-test was used to calculate the significance of changes in knowledge immediately after the webinar and comfort level with topics 35 days after the webinar.

**Results:**

Two hundred and sixty six participants from 10 SSA countries attended the first webinar, a series of six lectures on IHC theory, methods, and practice. Ninety-five participants from nine SSA countries provided a baseline assessment of pathology capacity and feedback. Mean knowledge increased by 17.4% immediately after the webinar (from 41.8% pre-webinar to 59.2% post, *p* =  < 0.0001). Self-reported comfort level in topics 35 days after the webinar increased by 11.3%, but this was not statistically significant (mean 3.36 pre- to 3.74 post, *p* = 0.1). Over six months, recordings were accessed 412 times. After six months, the second webinar had 93 participants from eight SSA countries. Membership in the DMP increased from 64 to 172; recordings were viewed 412 times in six months; and 113 participants from nine SSA countries completed surveys. Among 74 respondents who perform IHC, 43.5% reported moderate or significant positive practice changes such as improved antigen retrieval techniques and optimization of preanalytical variables. Over half (52.7%, *n* = 39) reported the quality of slides had moderately or significantly improved. After one year, a third webinar had 98 participants from eight SSA countries. Thirty-eight completed surveys, DMP membership increased to 199, and 1 reported launching IHC in a lab in Nigeria.

**Conclusions:**

Our program 1) reached hundreds of participants and provided a baseline assessment of pathology capacity across nine SSA countries; 2) created a novel mechanism to build pathology capacity and assess progress with this cohort; and 3) improved practices and the preparation of slides for over half performing manual IHC. After one year, interest was sustained. Tracking impact on diagnosis and treatment of BC in the region is needed long-term.

**Supplementary Information:**

The online version contains supplementary material available at 10.1186/s12885-023-11756-8.

## Background

Breast cancer is the most common malignancy worldwide, with highest mortality in sub-Saharan Africa (SSA) [1, 2]. Pathology capacity is essential to accurately classify breast cancer, and immunohistochemistry (IHC) is a methodology to identify the drivers of tumor growth and proliferation. A well-recognized need exists in SSA to build a skilled pathology workforce and strengthen pathology capacity [3]. Thus, we implemented a program to train SSA pathology technicians in high-quality manual immunohistochemistry (IHC).

IHC is a multistep process of tissue analysis; steps include fixation of tissue to stop degradation once it is removed from the patient, tissue processing to expose cellular antigens, and staining with antigen-specific antibodies. These biomarkers include estrogen receptor (ER), progesterone receptor (PR), human epidermal growth factor receptor 2 (HER2/neu), and Ki-67 (a marker of cellular proliferation), which are used to guide treatment decisions. While the process is automated in many parts of the world, the required equipment is often too expensive, too difficult to maintain, or simply unavailable in low-resource settings [4–7].

In our work to build pathology capacity in Hawassa, Ethiopia, we recognized that having laboratory technicians involved, motivated, and respected for their crucial role in tissue processing was essential to assure programmatic success. We observed barriers to accessing educational resources (e.g., travel restrictions due to COVID-19, local political instability, and training expense) that are frequently faced in low-resource settings. In response, we developed a free, long-term quality improvement project to generate additional networks of support and educational opportunities, such as the ability to partner with other institutions and stakeholders, apply for funding, and acquire equipment and reagents.

The aims of this quality improvement project were to 1) assess pathology capacity in SSA and address unmet educational needs of laboratory technicians; 2) create networks of professional contacts for mentorship while also creating a framework for tracking improvements and changes regionally over time; and 3) identify low-cost opportunities to improve practices that would benefit breast cancer diagnosis and treatment long-term. Here we report on the 1-year outcomes of this quality improvement project.

## Methods

We designed and implemented a quality improvement intervention with the overall goal to improve pathology capacity by training laboratory technicians in SSA. To overcome barriers of geography, expense, and lack of access to specialists, our free education intervention harnessed technological advances in remote conferencing, networking, and training. All tools of communication were selected for their accessibility to participants in low resource settings. Webinars were held on Zoom Webinar with prior registration requirement to enable tracking of attendance. Lecture materials (such as lecture slides and YouTube links to lecture recordings) were shared with participants by email. Surveys with 5-point Likert scale and free text were administered through Google Forms. The digital mentorship platform was an invitation-only Workplace group. A paired t-test was used to calculate the significance of changes in knowledge before and after the webinar and comfort level in topics 35 days after the webinar.

### Context

The intervention was designed for the target audience of histotechnicians and other pathology teams in sub-Saharan Africa. The project activities were announced to a network of professionals in pathology laboratories across 11 SSA countries by drawing on faculty and stakeholder regional contacts.

### Intervention

The intervention consisted of 1) developing relevant content for webinar-based lectures; 2) creating a platform for continual digital mentorship, networking, and sharing digital educational materials; and 3) periodic assessments of evolving pathology capacity, educational needs, practices changes, and quality improvements**.** In total, three interactive webinars were developed over one year with both didactic content and practical lectures from African experts with experience in IHC. One pre and post-knowledge assessment and four surveys were developed and administered. All were designed to be voluntary and not to be inconvenient or burdensome.

The program was initiated in January 2022 with an invitation to professionals to join a free webinar on theory and practice of IHC. A total of 374 professionals registered and were invited for all project activities for one year duration. The invitation provided a link to register for a webinar and to submit a pre-course baseline survey of pathology capacity and knowledge of IHC. The first webinar consisted of a series of six lectures with interactive question and answer (Q&A) sessions. Lecture content was based on Bancroft's Theory and Practice of Histological Techniques, 8th Edition. The lecture slides, recordings, and written Q&A answers were provided to registrants via email and links to Google Drive and YouTube. Two multiple choice questions per lecture were administered pre- and post-webinar to assess gains in knowledge. The Zoom webinar platform tracked participation and certificates were given to those who attended the webinar.

Thirty-five days following the first webinar, the 374 contacts were invited to join a digital mentorship platform (DMP) and submit a 35-day survey. The DMP was launched and faculty intermittently posted discussions and shared protocols to stimulate participant engagement.

After six months, the 374 contacts were invited to register for the 6-month webinar and encouraged to submit a 6-month survey to assess their comfort with IHC topics and perceptions of practice changes, institutional changes, and evolving education needs. During the webinar, participants were encouraged to join the DMP, submit a 6-month survey, and to attend the upcoming 1-year webinar with an opportunity to present their own experience with IHC.

**Fig. 1:**
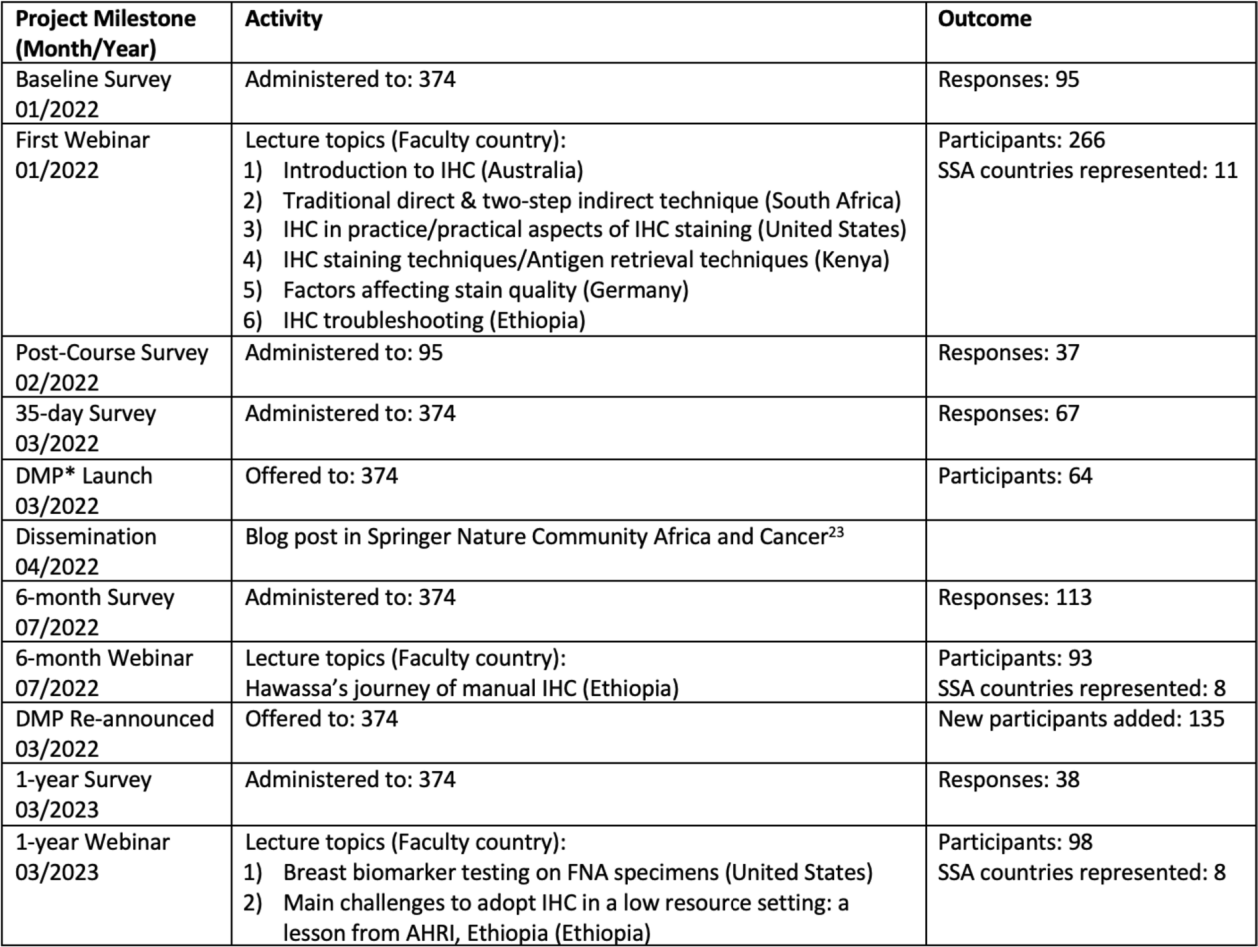
Timeline of project milestones, activities and outcomes. *DMP = Digital Mentorship Platform

After one year, the 374 contacts were invited to register for the anniversary webinar and take a short 1-year survey on progress and educational needs. Qualitative and quantitative data was collected between January 2022 and March 2023 (Fig. 1).

### Study of the intervention

**Fig. 2:**
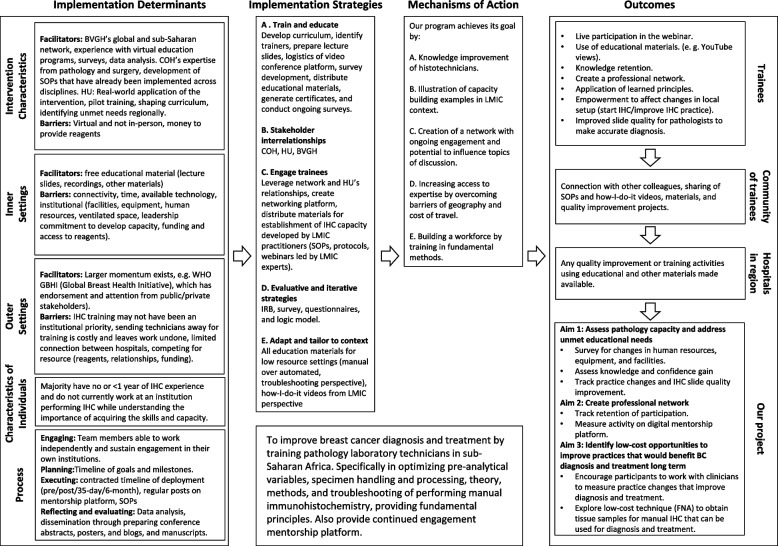
Implementation Research Logic Model. Logic model providing the framework for the intervention and tracking of outcomes

We used Implementation Research Logic Model (IRLM) guidelines to design a logic model that provided a framework for the intervention and tracking of outcomes (Fig. 2) [8]. With this approach, we measured the impact on individual participants, impact on the community of African technicians, programmatic changes within participating institutions, and the implementation of the project as a whole.

### Measures

We collected descriptive qualitative and quantitative information about the setting and cohort. Baseline survey questions assessed current practices, equipment, and available human resources in breast cancer treatment and pathology services in SSA [9]. Information was collected on the most common specimens processed, availability of IHC, use of biomarkers, and available treatments for breast cancer. Descriptive information of participants was collected, including country of origin, profession, years of IHC experience, comfort level in IHC, role in cancer treatment, and educational needs. Those who had registered for the webinar were provided with an assessment pre- and immediately post-webinar to ASSESS knowledge gains.

Voluntary follow-up surveys were developed for 35-day, 6-month, and 1-year time points and administered to the cohort of 374 professionals. Specifically, the 35-day survey asked participants to describe their perceptions on: whether the webinar content and education materials were useful and helpful; their comfort level with manual IHC, their education goals, and preferred device to access digital education materials; and their motivation to connect with other professionals, change practices in their institutions, and train others. The 6-month survey asked whether participants noticed any positive changes in their own practice or at their institutions, including whether participants perceived improvement in the quality of their slides. The 1-year survey asked participants to identify their top three topics interest for future educational webinars (Supplementary Materials & Methods).

Quantitative metrics were collected by tracking webinar registration, webinar attendance, numbers of times lecture recordings were viewed, and activity on the DMP.

### Analysis

Duplicate responses, incomplete entries, or entries of "not applicable" were removed during analysis of knowledge assessments or survey questions. Statistical analysis was performed using Paired t-test to measure changes in knowledge and comfort level in topics.

### Ethical considerations

We received IRB exemption from City of Hope as a quality improvement project. The project rationale, methods, knowledge assessments and survey questions received exemption prior to the intervention. Voluntary responses were de-identified.

## Results

**Fig. 3:**
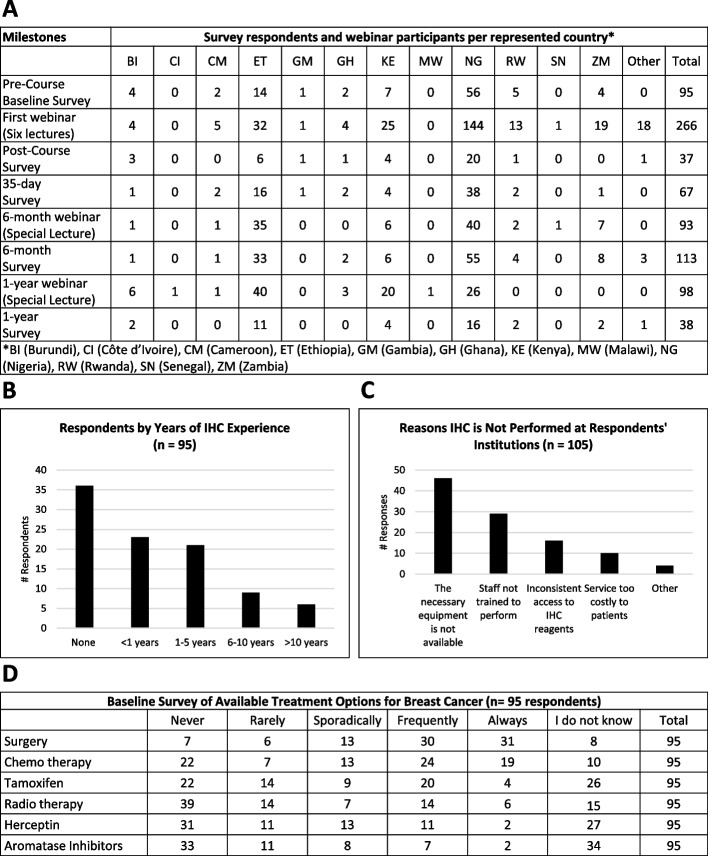
Participants and baseline survey responses. **A** Number of survey respondents and webinar participants from sub-Saharan African (SSA) countries. **B** Years of IHC experience of respondents to the pre-course baseline survey. **C** Main perceived barriers to institutions' lack of IHC capacity. **D** Most commonly available and most commonly unavailable treatments for breast cancer patients

A total of 374 registrants from 11 SSA countries registered for the program. The first webinar was launched in January 2022, with 266 participants from Burundi, Cameroon, Ethiopia, Gambia, Ghana, Kenya, Nigeria, Rwanda, Senegal, Tanzania, and Zambia attending (Fig. 3).

We surveyed registrants to understand the current status of IHC capacity in their region, as well as current practices, equipment, and human resources. A total of 100 registrants submitted the pre-course baseline survey (Fig. 3A). Five duplicate entries were removed. Of the remaining 95 respondents, most (*n* = 56) were from Nigeria. The majority (65.3%, *n* = 62) were histotechnicians. Other specialties included 11 pathologists, five residents, and 17 "other" healthcare professionals. Sixteen respondents (16.8%) had > 6 years of professional experience, yet the majority of respondents (*n* = 59, 62.1%) had little experience with IHC. Specifically, 23 (24.2%) had less than one year, and 36 (37.9%) had no experience (Fig. 3B).

We surveyed institutional plans to develop capacity for IHC and current capacity for breast cancer treatment. Of those whose institutions performed IHC, 34 (82.9%) performed it manually. Only two individuals stated that their institutions performed automated IHC, and five reported that they performed both manual and automated IHC. Notably, 43% reported that their institution planned to implement IHC within the next one to two years. When asked why their hospital lacked IHC capacity, the top four perceived barriers were lack of necessary equipment (*n* = 46), followed by lack of trained staff (*n* = 29), inconsistent access to IHC reagents (*n* = 16), and the high financial burden to patients (*n* = 10) (Fig. 3C).

Participants reported that the most common malignancies processed in their pathology laboratories were breast, followed by cervical, prostate, colorectal, gastric, blood, and liver. The most common IHC assay was for breast cancer biomarkers: estrogen receptor (100%); progesterone (92.5%); and Her2neu (87.5%). The most common treatments frequently or always available for patients with breast cancer were surgery (64.2%), chemotherapy (45.3%), endocrine therapy (34.8%), radiation (15%), and Her2neu directed therapy (< 14%). Notably, only two participants reported trastuzumab was always available (Fig. 3D).

Prior to the webinar, participants were asked about their perceptions of their role in oncology care. Ninety-eight percent perceived their role was extremely or very important in the overall care of cancer patients. The pre-course baseline survey revealed that the topics of most interest were: how to scale up pathology laboratory capacity; laboratory set-ups in other countries; specimen handling and analysis; breast cancer diagnosis and medical treatment; and breast cancer surgery and specimen handling in the operating room.

During the first webinar, a total of 266 participants attended at least one day of lectures and received a certificate. After the webinar, the mean knowledge assessment scores increased by 17.4% (from 41.8% pre to 59.2% post, *p* =  < 0.0001). Self-reported comfort level in topics 35 days after the webinar increased by 11.3%, but this was not statistically significant (mean 3.36 pre- to 3.74 post, *p* = 0.1). A post-course survey with 37 respondents found that 43% preferred using laptops and 41% mobile devices. The majority stated that they felt comfortable using a social media platform for professional networking and sharing of educational materials—32% extremely comfortable and 49% very comfortable. Six months after the webinar, the lecture recordings had been viewed 412 times. Sixty-four participants joined the DMP, using the platform to access posts of educational materials and discussions.

Six months into the program, a pathologist from Hawassa, Ethiopia, presented a lecture on his experience implementing manual IHC. The lecture described his training of surgical nursing teams in optimizing pre-analytical variables, protocol development, and lessons learned for areas of improvement. It is notable that within one month after the webinar, the video recording was accessed > 33 times and the DMP membership increased from 64 to 172. We posted slides of the lecture, the SOPs for manual IHC of estrogen and progesterone receptors using a microwave, and the protocol for making 10% neutral buffered formalin.

**Fig. 4:**
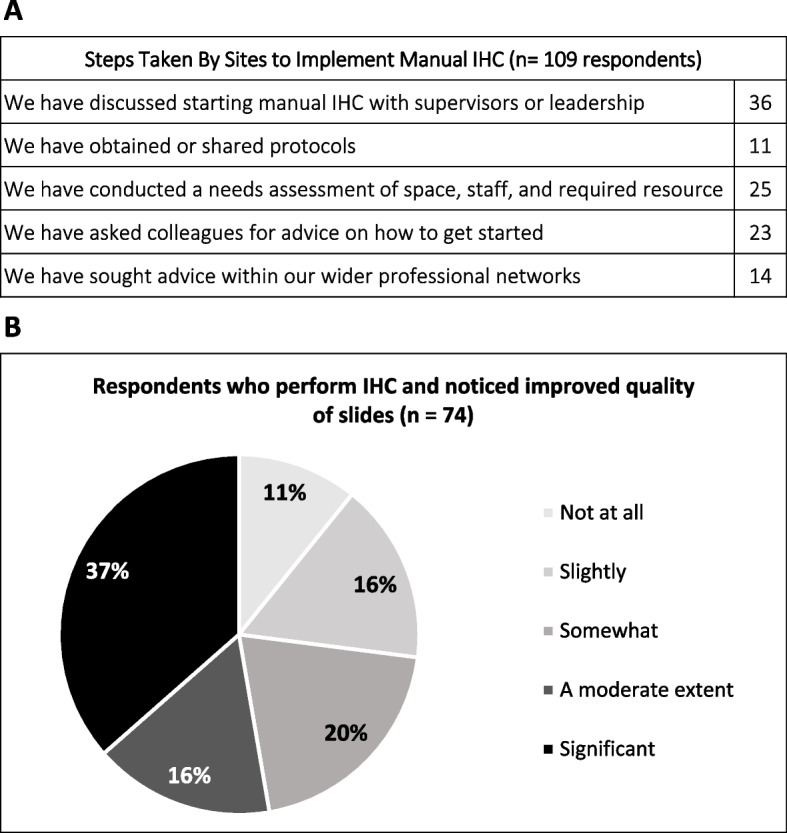
Positive changes in practice and quality improvement after 6 months. **A** Steps taken by survey respondents to implement manual IHC at their institutions. **B** Quality improvements noticed by respondents who perform IHC

A 6-month survey was administered, with 113 participants from nine SSA countries responding. In this cohort, 13% had neither attended the first webinar nor had joined the DMP at the time of the survey. One third of the survey respondents (32%) stated they had made changes in their local set-up to improve, implement, or scale-up IHC. Of the 6-month survey respondents who had not performed IHC, the majority stated that they had discussed starting it with hospital administration (64.3%, *n* = 36). Among the 74 respondents who perform IHC, 43.5% reported moderate or significant positive practice changes such as improved antigen retrieval techniques, protocol development, and training others on optimization of pre-analytical variables. More than half who perform IHC (52.7%, *n* = 39) reported the quality of slides had moderately or significantly improved (Fig. 4).

**Fig. 5:**
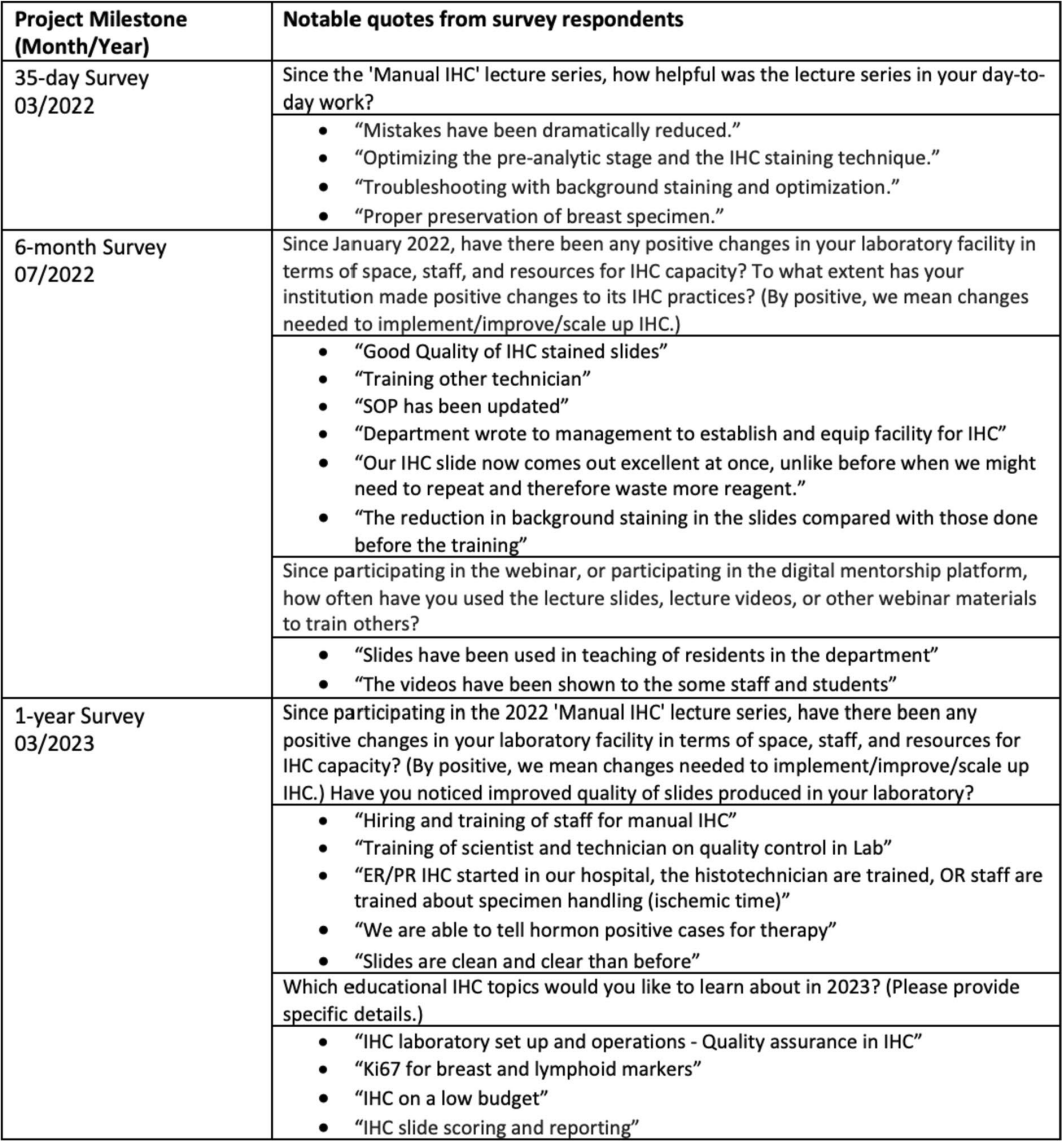
Notable quotes from participants at 6-month and 1-year time points. Unedited quotes submitted in surveys; no text has been modified

The 1-year webinar covered preparation of fine needle aspirate samples for IHC and the launching of IHC in Addis Ababa, Ethiopia. The 1-year webinar was attended by 98 participants from eight SSA countries. Thirty-eight individuals completed a 1-year survey, with many remarking on the high importance and relevance of the topic. Respondents were provided free text to identify areas of perceived need for education materials and webinars. One participant posted on the DMP about launching IHC in a laboratory in Nigeria (Fig. 5).

## Discussion

Our education program reached hundreds of participants in the pathology laboratory workforce in SSA and provided a baseline assessment of capacity in institutions in nine SSA countries. Our intervention was designed for the target audience of immunohistochemical technicians with little to no experience with manual IHC. Our survey of participants revealed that we successfully targeted the intended audience. We created a novel mechanism to build pathology capacity and assess progress with this cohort. We have sustained interest in the project in that we consistently invited 374 professionals who registered for the first webinar to participate in subsequent activities: nearly 100 participated in each webinar and membership in the DMP increased over one year duration.

Our findings demonstrate that breast cancers are the most common specimens being processed in our cohort, which aligns with reports that breast cancer is the most common malignancy in SSA and worldwide [1, 2, 10]. While the idea of using “leapfrog” technologies such as RT-qPCR (real-time quantitative reverse transcription polymerase chain reaction) has been proposed for the classification of breast cancer phenotypes, such technologies present their own challenges in terms of cost-effectiveness and availability. Thus, a well-trained workforce capable of performing manual IHC is still needed in low-income settings.

The optimization of pre-analytical variables for tissue processing is a fundamental step that requires training and involvement of multiple health care providers to ensure accurate analysis, regardless of the technique used. Training should be developed for the target audience of multidisciplinary teams, including surgeons, surgical nursing, and pathology technicians, with emphasis on the importance of developing SOPs appropriate for the local set up and workflows. We emphasized this topic in both the introductory and the 6-month webinar. Participants were provided lecture content and materials to disseminate the knowledge and train others in their institutions. Tracking is needed to measure how many others were subsequently trained and whether the training resulted in practice changes, such as limited cold ischemia time and appropriate fixation of specimen. Notably, reports that the quality of slides have improved suggest quality improvements overall, which may also include optimization of specimen handling and processing.

At the baseline assessment, only 14.9% of respondents reported running assays for Ki-67, although Ki-67 expression levels can inform expected sensitivity to chemotherapy and can help differentiate between luminal A and luminal B breast cancers. In the absence of multigene molecular prognostic and predictive tests, which are generally unavailable in SSA, Ki-67 expression could be helpful for effectively allocating scarce resources. Understanding the true distribution of breast cancer phenotypes among populations would help with allocation of resources associated with treatment (chemotherapy, endocrine therapy) and anticipated costs, human resources, and equipment. In settings with limited resources, prioritization of which reagents to purchase to perform IHC must account for the clinically useful and actionable information it will provide the clinician in making treatment decisions [11–13].

We observed that about one third of the reagents used in pathology laboratories performing IHC were for HER2 testing, although only two respondents reported that trastuzumab (Herceptin) was always available. HER2-directed therapies are expensive and are typically administered via intravenous infusion for at least one year. The patent for HER2 directed therapy has recently expired, thus allowing for cheaper manufacturing and therefore wider availability of this medication for patients in low-resource settings. Nevertheless, the facilitators and barriers to providing HER2 directed therapy to patients in SSA (including acceptability, accessibility, feasibility, and financial toxicity) are understudied. Therefore, when developing manual IHC programs, multidisciplinary discussions involving pathology, medical, and surgical oncology teams are critical in overall program planning and phased implementation, with prioritization of disease focus and selective purchase of reagents to align with available treatment resources [14–16].

In addition to the need for building capacity to perform manual IHC on surgically excised specimens, there is also a clinical need to perform manual IHC on fine needle aspirations (FNA) specimens, especially for patients presenting with locally advanced breast cancer in resource limited settings. FNA is a cost effective and minimally invasive method of tissue sampling that is widely available in SSA. Many patients with breast cancer in SSA present with locally advanced disease and would benefit from prompt, triaged delivery of neoadjuvant treatments or treatment for metastatic disease. Access to biomarker expression information via IHC performed on FNA samples would facilitate rational, timely, and cost-effective delivery of therapy to these patients. For example, patients with locally advanced disease whose tumors expressed ER or PR could be prescribed oral endocrine therapy while treatments such as chemotherapy or surgery are being arranged. The 1-year webinar covered the practical aspects of preparing samples from FNA for manual IHC in resource limited settings. The interactive webinar was attended by 98 participants from eight SSA countries, many of whom remarked on the high importance and relevance of the topic [17–22].

It is notable that 98% of participants in our survey perceived the role of histotechnicians in cancer care as extremely and very important. This reflects attitudes of a workforce with intrinsic motivation and capacity to exceed expectations. We aimed to design an ongoing mentorship program that supports, engages, and motivates a dedicated workforce to perform IHC manually in a precise and timely manner in order to obtain reliable results that directly impact patient care. Our program recognized histotechnicians’ important roles as vital to improving the capacity and quality of pathology services in SSA.

To our knowledge, this program created a new professional network of its kind in the region with an approach focused on sustaining interest and relevance. Our faculty intermittently provide posts and questions to stimulate discussion and keep the momentum of interest sustained. Participants were surveyed about their education needs and were invited to share their experience with IHC in webinars and the DMP to encourage engagement in the program [23]. Thus, our education program created a novel mechanism to build pathology capacity in SSA and assess progress with this cohort.

### Limitations and strengths

We acknowledge that people learn in different ways, and that knowledge gain and comfort level with topics is not perfectly measured through pre- and post-lecture assessments, which may be subjective.There are well known limitations to knowledge acquisition and retention via passive participation in web-based lectures. Thus, we designed an education intervention that was flexible and utilized multiple modalities. It enabled participants to access content at different times of the day, or when it was useful for them in practical application. While we cannot precisely quantify total knowledge gain or changes in comfort level, we regard the self-reported improvements in laboratory quality following participation in our education program as evidence of success. For instance, six months following the initial webinar, most respondents performing IHC reported improvements in slide quality. A possible objective metric for program effectiveness would be external review of slides, which is in our plans for future capacity building projects and was beyond the scope of this education intervention. Such improvements ultimately translate to better patient care via better pathologic diagnoses and more accurately tailored therapy. Continued reinforcement of knowledge was within the scope of this digital education intervention in the form of participatory webinars, lecture recordings, lecture presentations, and engagement on the DMP with faculty. However, reinforcement of skills was beyond the scope of this project as it would have required hands-on training with access to equipment, consumables, reagents, and materials that this program did not provide.

## Conclusions

Our education intervention 1) reached hundreds of participants and provided a baseline assessment of pathology capacity in institutions in nine sub-Saharan African countries; 2) created a novel mechanism to build pathology capacity and assess progress with this cohort; and 3) improved practices and the preparation of slides for over half of those performing manual IHC. Sustained engagement is needed to increase capacity. Tracking of improvements in diagnosis and treatment of patients in the region is needed.

### Supplementary Information

**Additional file 1.**

## Data Availability

The datasets used to generate these results in the current study are available from the corresponding author on reasonable request.

## References

[1] Sung H, Ferlay J, Siegel RL, Laversanne M, Soerjomataram I, Jemal A (2021). Global Cancer Statistics 2020: GLOBOCAN Estimates of Incidence and Mortality Worldwide for 36 Cancers in 185 Countries. CA Cancer J.

[2] Anderson BO, Ilbawi AM, Fidarova E, Weiderpass E, Stevens L, Abdel-Wahab M, Mikkelsen B (2021). The Global Breast Cancer Initiative: a strategic collaboration to strengthen health care for non-communicable diseases. Lancet Oncol.

[3] Martei YM, Pace LE, Brock JE, Shulman LN (2018). Breast cancer in low- and middle-income countries: why we need pathology capability to solve this challenge. Clin Lab Med.

[4] Mugabe M, Ho KE, Ruhangaza D, Milner D, Rugwizangoga B, Chu VC, Wu NC, Rizo A, Weidler JM, Wong W, Bates M (2021). Use of the Xpert breast cancer STRAT4 for biomarker evaluation in tissue processed in a developing country. Am J Clin Pathol.

[5] Patel K, Chumba D, Strother RM, Ndiangui F, Jacobson W, Dodson C, Strate RW, Smith JW, Resnic MB (2016). Development of immunohistochemistry services for cancer care in western Kenya: implications for low-and middle-income countries. Af J Lab Med.

[6] Karim S, Sunderji Z, Jalink M, Mohamed S, Mallick I, Msadabwe-Chikuni SC, Delgarno NJ, Hammad N, Berry S (2021). Oncology training and education initiatives in low and middle income countries: a scoping review. Ecancermedicalscience.

[7] Ngwa W (2022). Cancer in sub-Saharan Africa: a Lancet Oncology Commission. Lancet Oncology.

[8] Smith JD, Li DH, Rafferty MR (2020). The implementation research logic model: a method for planning, executing, reporting, and synthesizing implementation projects. Implement Sci.

[9] Binagwaho A, Wagner CM, Farmer PE (2016). A vision for global cancer medicine: pursuing the equity of chance. J Clin Oncol.

[10] McCormack V, McKenzie F, Foerster M, Zietsman A, Galukande M, Adisa C, Anele A, Parham G, Pinder LF, Cubasch H, Joffe M, Beaney T, Quaresma M, Togawa K, Abedi-Ardekani B, Anderson BO, Schüz J, Dos-Santos-Silva I (2020). Breast cancer survival and survival gap apportionment in sub-Saharan Africa (ABC-DO): a prospective cohort study. Lancet Glob Health.

[11] Kantelhardt EJ, Mathewos A, Aynalem A, Wondemagegnehu T, Jemal A, Vetter M, Knauf E, Reeler A, Bogale S, Thomssen C, Stang A, Gemechu T, Trocchi P, Yonas B (2014). The prevalence of estrogen receptor-negative breast cancer in Ethiopia. BMC Cancer.

[12] Desalegn Z, Yohannes M, Porsch M, Stückrath K, Anberber E, Santos P, Bauer M, Addissie A, Bekuretsion Y, Assefa M, Worku Y, Abebe T, Taylor L, Kantelhardt E, Vetter M (2022). Intrinsic subtypes in Ethiopian breast cancer patient. Breast Cancer Res Treat.

[13] Taylor L, Harris C, Abebe T, Addissie A, Assefa M, Kantelhardt EJ (2021). A decade of strengthening breast oncology in Ethiopia. Lancet Oncol.

[14] Yerakly F, Tadele AK (2022). Histopathologic patterns of breast lesions in Hawassa University Comprehensive Specialized Hospital, Sidama Region, Ethiopia: a six-year retrospective study (September 2015 G.C to August 2020 G.C.). Clin Oncol..

[15] Okubazgi G, Berhane B, Nigussie M, Tsegaye A, Hassen F (2020). Status of Histopathology Services in Ethiopia. Am J Clin Pathol.

[16] Global Breast Cancer Initiative Implementation Framework: assessing, strengthening and scaling-up of services for the early detection and management of breast cancer. Geneva: World Health Organization; 2023. Licence: CC BY-NC-SA 3.0 IGO. https://www.who.int/initiatives/global-breast-cancer-initiative.

[17] Taye E, Ali MM, Toma A, Teklehaimanot A (2022). Comparison of Fine Needle Aspiration Cytology and Histopathology in the diagnosis of lymph node pathologies at health facilities located in Hawassa: A 5-year retrospective study. SAGE Open Med.

[18] Ayele W, Addissie A, Wienke A, Unverzagt S, Jemal A, Taylor L, Kantelhardt EJ (2021). Breast awareness, self-reported abnormalities, and breast cancer in rural Ethiopia: a survey of 7,573 women and predictions of the national burden. Oncologist.

[19] Trabitzsch J, Wondimagegnehu A, Afework T, Stoeter O, Gizaw M, Getachew S, Feyisa JD, Taylor L, Wienke A, Addissie A, Kantelhardt EJ. Pathways and Referral of Patients with Cancer in Rural Ethiopia: A Multi-center Retrospective Cohort Study. Oncologist. 2023;28(6):e359–e368. 10.1093/oncolo/oyad032. 10.1093/oncolo/oyad032 PMC10243765 36940294

[20] Tesfaw A, Getachew S, Addissie A, Jemal A, Wienke A, Taylor L, Kantelhardt EJ (2021). Late-stage diagnosis and associated factors among breast cancer patients in South and Southwest Ethiopia: a multicenter study. Clin Breast Cancer.

[21] Shita A, Yalew AW, Seife E, Afework T, Tesfaw A, Gufue ZH, Rabe F, Taylor L, Kantelhardt EJ, Getachew S (2023). Survival and predictors of breast cancer mortality in South Ethiopia: a retrospective cohort study. PLoS ONE.

[22] Gong Y, Symmans WF, Krishnamurthy S, Patel S, Sneige N (2004). Optimal fixation conditions for immunocytochemical analysis of estrogen receptor in cytologic specimens of breast carcinoma. Cancer Cytopathol.

[23] Taylor L, Schmolze D, Graef K, Setiawan L, Alem AT. Nature Portfolio Cancer Community. Improving Breast Cancer Diagnosis and Treatment by Training Pathology Technicians in sub-Saharan Africa. [Internet, published April 23, 2022]. Odedina B, editor. New York, NY: Springer Nature. 2022. Available from: https://go.nature.com/3vsm8Jy. [Cited 2022 May 10, 2022].

