# Supplementary material for: Building pathology capacity in sub-Saharan Africa to improve breast cancer diagnosis and treatment: training laboratory technicians in high-quality manual immunohistochemistry
Source: BMC Cancer. 2024 Jan 3;24:32. doi: 10.1186/s12885-023-11756-8 (PMC10763384; doi:10.1186/s12885-023-11756-8)
Supplement: Supplementary file 1 — Additional file 1. [file 12885_2023_11756_MOESM1_ESM.docx]

**A. Pre-Course Baseline Survey**

**A.1 Pre-Course Baseline**

1. Name
2. Email Address
3. Institution and Department
4. Country
5. Position/Profession
6. How many years of experience do you have in your profession?
   1. <1 years
   2. 1-5 years
   3. 6-10 years
   4. >10 years
7. How many years of experience do you have performing immunohistochemistry (IHC)?
   1. <1 years
   2. 1-5 years
   3. 6-10 years
   4. >10 years
   5. None
8. How many staff members work in the pathology lab at your hospital/institution?

|  | 1-3 | 4-6 | 7-10 | 11-15 | 16-20 | 21-25 | >25 | None | I do not know |
| --- | --- | --- | --- | --- | --- | --- | --- | --- | --- |
| Histotechnicians |  |  |  |  |  |  |  |  |  |
| Pathologists |  |  |  |  |  |  |  |  |  |
| Pathology residents |  |  |  |  |  |  |  |  |  |

1. Does your current hospital/institution perform IHC?
   1. Yes
   2. No
   3. No, but my hospital/institution plans to implement IHC in the next 1-2 years
   4. I do not know
   5. Not applicable
2. If your current hospital/institution does not perform IHC, why? Select all that apply.
   1. Staff not trained to perform IHC
   2. Service too costly to patients
   3. The necessary equipment is not available
   4. Inconsistent access to IHC reagents
   5. Other: _________________
   6. I don't know
   7. Not applicable
3. Which IHC methods does your hospital/institution use?
   1. Manual IHC
   2. Automated IHC
   3. Both manual and automated IHC
   4. None
   5. I do not know
4. If your hospital/institution performs IHC, which biomarkers do you test for (select all that apply)?
   1. HER2neu
   2. ER (Estrogen Receptor)
   3. PR (Progesterone Receptor)
   4. Ki-67
   5. I do not know
   6. We do not perform IHC at this time
   7. Other: _________________
5. How many cancer specimens does your laboratory process per month?

|  | 1-5 | 6-10 | 11-25 | 26-50 | 51-75 | >75 | None | I do not know, but can find out and can get back to you |
| --- | --- | --- | --- | --- | --- | --- | --- | --- |
| Breast |  |  |  |  |  |  |  |  |
| Colorectal |  |  |  |  |  |  |  |  |
| Gastric |  |  |  |  |  |  |  |  |
| Prostate |  |  |  |  |  |  |  |  |
| Cervical |  |  |  |  |  |  |  |  |
| Liver |  |  |  |  |  |  |  |  |
| Blood |  |  |  |  |  |  |  |  |

1. Please note the availability of treatment options for breast cancer patients at your hospital/institution.

|  | Never | Rarely | Sporadically | Frequently | Always | I do not know, but can find out and get back to you |
| --- | --- | --- | --- | --- | --- | --- |
| Herceptin |  |  |  |  |  |  |
| Tamoxifen |  |  |  |  |  |  |
| Aromatase inhibitors |  |  |  |  |  |  |
| Chemotherapy |  |  |  |  |  |  |
| Radiation |  |  |  |  |  |  |
| Surgery |  |  |  |  |  |  |

1. On a scale of 1-5, please describe your comfort level with the below topics.

|  | Not comfortable at all | Slightly comfortable | Somewhat comfortable | Very comfortable | Extremely comfortable |
| --- | --- | --- | --- | --- | --- |
| How to optimize pre-analytic variables for IHC |  |  |  |  |  |
| Manual staining techniques |  |  |  |  |  |
| Factors affecting staining quality |  |  |  |  |  |
| Trouble shooting problems in manual IHC |  |  |  |  |  |
| Antigen removal techniques |  |  |  |  |  |
| Breast cancer and its treatment |  |  |  |  |  |

1. On a scale of 1 to 5, how important would you rank the role of histotechnicians in cancer care?
   1. 1 - Not important at all
   2. 2 - Slightly important
   3. 3 - Somewhat important
   4. 4 - Very important
   5. 5 - Extremely important
2. In your opinion, what is the role of histotechnicians in the care of cancer patients?
   1. _____________________________
3. On a scale of 1 to 5, please rank your interest level in other topics for continuing education.

|  | 1 - Not interested at all | 2 - Slightly  interested | 3 - Somewhat interested | 4 - Very interested | 5 - Extremely  interested |
| --- | --- | --- | --- | --- | --- |
| Breast Cancer diagnosis and medical treatment |  |  |  |  |  |
| Breast cancer surgery and specimen handling in the operating room |  |  |  |  |  |
| Specimen handling and analysis |  |  |  |  |  |
| Pathology lab setups in other countries |  |  |  |  |  |
| How to scale up pathology lab capacity |  |  |  |  |  |

**A.2 Pre-Course Knowledge Assessment**

1. Select which of the following statements are true about IHC. Select all that apply.
   1. IHC methods allow identification of cellular epitopes in tissue that has been formalin fixed and paraffin wax processed.
   2. Many antibodies are able to identify tissue epitopes (or antigens) that survive formalin fixation and paraffin wax processing.
   3. In order to stain tissue, these antigens must be unmasked.
   4. None of the above
   5. I do not know
2. Manual methods of unmasking antigens can be performed in a variety of ways. Select all that apply.
   1. Proteolytic
   2. Microwave oven irradiation
   3. Pressure cooker heating
   4. Water bath heating
   5. Steamer heating
   6. None of the above
   7. I do not know
3. The selection of IHC staining technique to detect tissue antigens depends upon the type of specimen, the degree of sensitivity required, and the type of tissue preparation. Which type of tissue preparation can be used for IHC? Select all that apply.
   1. Frozen sections
   2. Paraffin wax sections
   3. Resin sections
   4. Cytological preparations
   5. None of the above
   6. I do not know
4. Select which of the following statements are true about two step indirect technique. Select all that apply.
   1. Commonly uses horseradish peroxidase with a chromogen substrate
   2. Is more sensitive than the traditional direct technique because multiple secondary antibodies react with different sites on the primary antibody to enhance the signal
   3. Can be used for analysis of prostate and ovarian cancers
   4. None of the above
   5. I do not know
5. High quality IHC is important because it is used to classify breast cancer subtypes which helps help doctors make treatment decisions for patients. How much do you agree with this statement?
   1. Strongly agree
   2. Agree
   3. Neutral
   4. Disagree
   5. Strongly disagree
6. Optimizing pre-analytic variables includes several steps. Select all that apply.
   1. Ensuring and documenting a cold ischemia time of less than 1 hour
   2. Slicing large specimen before fixation
   3. Placing specimen in appropriate formalin volume
   4. Selection of the appropriate specimen container
   5. Ensuring appropriate time in formalin (6-72 hours)
   6. Appropriate formalin fixation
   7. Appropriate paraffin embedding
   8. None of the above
   9. I do not know
7. Which of these is true? Select all that apply.
8. Each method of antigen unmasking can differ per laboratory.
9. Optimal methods of unmasking need to be evaluated using the laboratory's own material.
10. Digestion or heating times may need to be modified depending on local conditions.
11. Techniques in IHC staining involve sequential incubations in antibodies and labeling systems separated by washes in buffer.
12. All the above are true.
13. I do not know
14. When preparing manual IHC, it is essential to ensure several steps. Select all that apply.
    1. Each antibody is used at an appropriate dilution.
    2. Antibodies do not evaporate during incubation.
    3. Each antibody is completely removed with washes if unbound before the next specific antibody or reagent is added.
    4. Antibody incubations are carried out in a humid atmosphere.
    5. None of the above
    6. I do not know
15. Assuring optimal formalin fixation involves several steps. Select which steps must be taken.
16. Putting tissue in formalin as soon as possible to limit cold ischemia time to <1 hour.
17. Tissue thickness of a "bread loafed” large specimen should not be thicker than 2 cm.
18. The tissue to formalin ratio should be about 1:10.
19. Formalin should be PBS buffered and methanol stabilized (10% formalin = 4% formaldehyde) and never reused.
20. Fixation should occur for 8-48 hours at room temperature.
21. None of the above
22. I do not know
23. Assign numbers 1 to 5 to these IHC staining steps in the correct order of their sequence (1 = first step, 2 = second step, 3 = third step, 4 = fourth step, 5 = last step)
    1. Tissue preparation (limited ischemia time, optimal formalin fixation and paraffin embedding): _____
    2. Deparaffinization of freshly sliced tissue sections involves heating slides for at least 30 minutes (60-62˚ C) followed by incubation times of 3 minutes with fresh solutions (could be xylene, 100% Ethanol, 50% Ethanol, or water): ______
    3. Peroxidase inactivation: ______
    4. Antigen retrieval using either heat methods or enzymatic antigen retrieval methods: _____
    5. Blocking, primary antibody incubation, secondary antibody incubation, DAB: ____
    6. I do not know
24. Washing is an important step in manual IHC. Which of the below statements are correct? Select all that apply
25. Should be repeated 3 times for optimal results.
26. Should be done with fresh washing solutions that should not be reused.
27. Is important because inadequate washing results in non-specific staining or background staining.
28. None of the above
29. I do not know

1. Which of the following is true about the steps of paraffin embedding? Select all that apply.
2. Rinse out the formalin with a 50% alcohol solution, which should be changed once a week.
3. Dehydrating the tissue with acetone or 70% alcohol for >4 hours will make the tissue become too hard.
4. Xylene acts as a linking agent between alcohol and paraffin and as a clearing agent.
5. Imbue tissue with paraffin of high quality.
6. None of the above
7. I do not know

**B. Post-Course Survey**

**B.1 Accessibility and learning preferences**

1. Name
2. Email Address
3. On a scale of 1 to 5, please rank your preference level for methods of learning new educational content.
4. Live lecture with Q&A
5. Review lecture slides
6. Watch video of lecture
7. Small group discussion
8. Read textbook
9. On a scale of 1 to 5, please rank the practicality of joining the ‘Manual IHC for Histotechnicians’ virtual lecture series zoom webinar from electronic devices.
10. My mobile device
11. My laptop computer
12. My desktop computer
13. Work computer
14. Sharing screen with a colleague/friend
15. On a scale of 1 to 5, please rank the practicality of accessing educational materials (lecture slides, lecture videos) from electronic devices.
16. My mobile device
17. My laptop computer
18. My desktop computer
19. Work computer
20. Someone else’s device
21. On a scale of 1 to 5, please rank your comfort level with using social media to engage with a professional network and exchange relevant resources, experiences, and educational materials.
22. 1 - Not comfortable at all
23. 2 - Slightly comfortable
24. 3 - Somewhat comfortable
25. 4 - Very comfortable
26. 5 - Extremely comfortable
27. On a scale of 1 to 5, please rank your interest level in other topics for continuing education.

|  | 1 - Not interested at all | 2 - Slightly interested | 3 - Somewhat interested | 4 - Very interested | 5 - Extremely interested |
| --- | --- | --- | --- | --- | --- |
| Breast Cancer diagnosis and medical treatment |  |  |  |  |  |
| Breast cancer surgery and specimen handling in the operating room |  |  |  |  |  |
| Specimen handling and analysis |  |  |  |  |  |
| Pathology lab setups in other countries |  |  |  |  |  |
| How to scale up pathology lab capacity |  |  |  |  |  |

**B.2 Post-Course Knowledge Assessment (same as Pre-Course Knowledge Assessment)**

1. Select which of the following statements are true about IHC. Select all that apply.
   1. IHC methods allow identification of cellular epitopes in tissue that has been formalin fixed and paraffin wax processed.
   2. Many antibodies are able to identify tissue epitopes (or antigens) that survive formalin fixation and paraffin wax processing.
   3. In order to stain tissue, these antigens must be unmasked.
   4. None of the above
   5. I do not know
2. Manual methods of unmasking antigens can be performed in a variety of ways. Select all that apply.
   1. Proteolytic
   2. Microwave oven irradiation
   3. Pressure cooker heating
   4. Water bath heating
   5. Steamer heating
   6. None of the above
   7. I do not know
3. The selection of IHC staining technique to detect tissue antigens depends upon the type of specimen, the degree of sensitivity required, and the type of tissue preparation. Which type of tissue preparation can be used for IHC? Select all that apply.
   1. Frozen sections
   2. Paraffin wax sections
   3. Resin sections
   4. Cytological preparations
   5. None of the above
   6. I do not know
4. Select which of the following statements are true about two step indirect technique. Select all that apply.
   1. Commonly uses horseradish peroxidase with a chromogen substrate
   2. Is more sensitive than the traditional direct technique because multiple secondary antibodies react with different sites on the primary antibody to enhance the signal
   3. Can be used for analysis of prostate and ovarian cancers
   4. None of the above
   5. I do not know
5. High quality IHC is important because it is used to classify breast cancer subtypes which helps help doctors make treatment decisions for patients. How much do you agree with this statement?
   1. Strongly agree
   2. Agree
   3. Neutral
   4. Disagree
   5. Strongly disagree
6. Optimizing pre-analytic variables includes several steps. Select all that apply.
   1. Ensuring and documenting a cold ischemia time of less than 1 hour
   2. Slicing large specimen before fixation
   3. Placing specimen in appropriate formalin volume
   4. Selection of the appropriate specimen container
   5. Ensuring appropriate time in formalin (6-72 hours)
   6. Appropriate formalin fixation
   7. Appropriate paraffin embedding
   8. None of the above
   9. I do not know
7. Which of these is true? Select all that apply.
8. Each method of antigen unmasking can differ per laboratory.
9. Optimal methods of unmasking need to be evaluated using the laboratory's own material.
10. Digestion or heating times may need to be modified depending on local conditions.
11. Techniques in IHC staining involve sequential incubations in antibodies and labeling systems separated by washes in buffer.
12. All the above are true.
13. I do not know
14. When preparing manual IHC, it is essential to ensure several steps. Select all that apply.
    1. Each antibody is used at an appropriate dilution.
    2. Antibodies do not evaporate during incubation.
    3. Each antibody is completely removed with washes if unbound before the next specific antibody or reagent is added.
    4. Antibody incubations are carried out in a humid atmosphere.
    5. None of the above
    6. I do not know
15. Assuring optimal formalin fixation involves several steps. Select which steps must be taken.
16. Putting tissue in formalin as soon as possible to limit cold ischemia time to <1 hour.
17. Tissue thickness of a "bread loafed” large specimen should not be thicker than 2 cm.
18. The tissue to formalin ratio should be about 1:10.
19. Formalin should be PBS buffered and methanol stabilized (10% formalin = 4% formaldehyde) and never reused.
20. Fixation should occur for 8-48 hours at room temperature.
21. None of the above
22. I do not know
23. Assign numbers 1 to 5 to these IHC staining steps in the correct order of their sequence (1 = first step, 2 = second step, 3 = third step, 4 = fourth step, 5 = last step)
    1. Tissue preparation (limited ischemia time, optimal formalin fixation and paraffin embedding): _____
    2. Deparaffinization of freshly sliced tissue sections involves heating slides for at least 30 minutes (60-62˚ C) followed by incubation times of 3 minutes with fresh solutions (could be xylene, 100% Ethanol, 50% Ethanol, or water): ______
    3. Peroxidase inactivation: ______
    4. Antigen retrieval using either heat methods or enzymatic antigen retrieval methods: _____
    5. Blocking, primary antibody incubation, secondary antibody incubation, DAB: ____
    6. I do not know
24. Washing is an important step in manual IHC. Which of the below statements are correct? Select all that apply
25. Should be repeated 3 times for optimal results.
26. Should be done with fresh washing solutions that should not be reused.
27. Is important because inadequate washing results in non-specific staining or background staining.
28. None of the above
29. I do not know

1. Which of the following is true about the steps of paraffin embedding? Select all that apply.
2. Rinse out the formalin with a 50% alcohol solution, which should be changed once a week.
3. Dehydrating the tissue with acetone or 70% alcohol for >4 hours will make the tissue become too hard.
4. Xylene acts as a linking agent between alcohol and paraffin and as a clearing agent.
5. Imbue tissue with paraffin of high quality.
6. None of the above
7. I do not know

**C. 35-day Survey**

**C.1 Networking and Motivation**

1. Name
2. Email Address
3. Institution and Department
4. Country
5. Position/Profession
6. How many years of experience do you have in your profession?
7. <1 years
8. 1-5 years
9. 6-10 years
10. >10 years
11. How many years of experience do you have performing immunohistochemistry (IHC)?
12. <1 years
13. 1-5 years
14. 6-10 years
15. >10 years
16. None
17. How many staff members work in the pathology lab at your hospital/institution?

|  | 1-3 | 4-6 | 7-10 | 11-15 | 16-20 | 21-25 | >25 | None | I do not know |
| --- | --- | --- | --- | --- | --- | --- | --- | --- | --- |
| Histotechnicians |  |  |  |  |  |  |  |  |  |
| Pathologists |  |  |  |  |  |  |  |  |  |
| Pathology residents |  |  |  |  |  |  |  |  |  |

1. Does your hospital/institution perform IHC to diagnose cancer patients?
2. Yes
3. No
4. I do not know
5. During and after the ‘Manual IHC for Histotechnicians’ lecture series, we shared educational materials with you. Please indicate which materials, if any, you have used. Select all that apply.
6. Attended live webinar day 1 (lecture 1+2)
7. Attended live webinar day 2 (lectures 3+4)
8. Attended live webinar day 3 (lecture 5+6)
9. Reviewed lecture slides
10. Watched video recordings of lectures
11. I did not use any of the shared materials
12. If you are interested in engaging in a virtual platform to promote professional networking and sharing of helpful materials, please indicate for which platform you would like to be registered with the email address you provided above.
13. Histotechnicians
14. Pathologists
15. Both histotechnicians and pathologists
16. Not interested - do not register me at this time
17. On a scale of 1 to 5, please indicate the current level at which you feel engaged with your institution’s goals to build capacity for cancer care.

a. 1 - Not engaged at all

b. 2 - Slightly engaged

c. 3 - Somewhat engaged

d. 4 - Very engaged

e. 5 - Extremely engaged

1. On a scale of 1 to 5, please indicate the current level at which you are professionally connected with other professionals in your field of expertise (histotechnology and/or pathology)*.

a. 1 - Not connected at all

b. 2 - Slightly connected

c. 3 - Somewhat connected

d. 4 - Very connected

e. 5 - Extremely connected

1. Please indicate which, if any, professional connections you can contact for advice or help. Select all that apply.

a. 1 - I do not have any professional connections

b. 2 - I have professional connections within my current institution/hospital

c. 3 - I have professional connections at institutions/hospitals outside my place of work

d. 4 - I have professional connections in other African countries

e. 5 - I have professional connections outside of Africa

1. If your institution/hospital currently performs IHC and you see need for improvement, how motivated do you feel to improve practices in your current setting?

a. 1 - Not motivated at all

b. 2 - Slightly motivated

c. 3 - Somewhat motivated

d. 4 - Very motivated

e. 5 - Extremely motivated

1. If your institution/hospital currently does not perform IHC, how motivated do you feel to advocate for establishing IHC capacity in your current setting, e. g. talk to leadership?

a. 1 - Not motivated at all

b. 2 - Slightly motivated

c. 3 - Somewhat motivated

d. 4 - Very motivated

e. 5 - Extremely motivated

**C.2 Follow-up**

1. Please describe your comfort level with the below topics.

|  | Not comfortable at all | Slightly comfortable | Somewhat comfortable | Very comfortable | Extremely comfortable |
| --- | --- | --- | --- | --- | --- |
| How to optimize pre-analytic variables for IHC |  |  |  |  |  |
| Manual staining techniques |  |  |  |  |  |
| Factors affecting staining quality |  |  |  |  |  |
| Trouble shooting problems in manual IHC |  |  |  |  |  |
| Antigen removal techniques |  |  |  |  |  |
| Breast cancer and its treatment |  |  |  |  |  |

1. On a scale of 1 to 5, please rank your interest level in other topics for continuing education.

|  | 1 - Not interested at all | 2 - Slightly interested | 3 - Somewhat interested | 4 - Very interested | 5 - Extremely interested |
| --- | --- | --- | --- | --- | --- |
| Breast Cancer diagnosis and medical treatment |  |  |  |  |  |
| Breast cancer surgery and specimen handling in the operating room |  |  |  |  |  |
| Specimen handling and analysis |  |  |  |  |  |
| Pathology lab setups in other countries |  |  |  |  |  |
| How to scale up pathology lab capacity |  |  |  |  |  |

1. Since the "Manual IHC for Histotechnicians" lecture series, how helpful was the lecture series in your day-to-day work?
2. 1 - Not helpful at all
3. 2 - Slightly helpful
4. 3 - Somewhat helpful
5. 4 - Very helpful
6. 5 - Extremely helpful
7. Not applicable
8. Please list any examples, even if minor: _____________________________
9. Since the "Manual IHC for Histotechnicians" lecture series, how often did you or your hospital/institution use what was taught in the "Manual IHC for Histotechnicians" virtual lecture series in your setup?
10. 1 - Never
11. 2 - Rarely
12. 3 - Sometimes
13. 4 - Often
14. 5 - Very often
15. Not applicable
16. Please list any examples, even if minor: _____________________________
17. Since the "Manual IHC for Histotechnicians" lecture series, how comfortable were you with performing manual IHC since the "Manual IHC for Histotechnicians" virtual lecture series?
18. 1 - Not comfortable at all
19. 2 - Slightly comfortable
20. 3 - Somewhat comfortable
21. 4 - Very comfortable
22. 5 - Extremely comfortable
23. Not applicable
24. Please list any examples, even if minor: _____________________________
25. Since the "Manual IHC for Histotechnicians" lecture series, how important was manual IHC in making treatment decisions for breast cancer patients at your institution?
26. 1 - Not important at all
27. 2 - Slightly important
28. 3 - Somewhat important
29. 4 - Very important
30. 5 - Extremely important
31. Not applicable
32. Please list any examples, even if minor: _____________________________
33. Since the "Manual IHC for Histotechnicians" lecture series, how often did you contact the organizers or a colleague center (e. g. another center that participated in the program) for help with a question related to IHC?
34. 1 - Never
35. 2 - Rarely
36. 3 - Sometimes
37. 4 - Often
38. 5 - Very often
39. Not applicable
40. Please list any examples, even if minor: _____________________________
41. Since your participation in "Manual IHC for Histotechnicians", have you participated in other training programs relevant to pathology?
42. Yes
43. No
44. If yes, please list training programs: _____________________________
45. Have you used the knowledge you learned in "Manual IHC for Histotechnicians" to train other colleagues within your institution?
46. Yes
47. No
48. Not applicable
49. If yes, please list how many colleagues you trained: _____________________________
50. Have you experienced any major challenges or obstacles impacting your ability to apply the knowledge gained during the "Manual IHC for Histotechnicians" virtual lecture series?

a. Yes

b. No

c. Not applicable

d. If yes, please list any examples, even if minor: _____________________________

**D. 6-month Survey**

**D.1 Follow-up**

1. Name
2. Email Address
3. Institution and Department
4. Country
5. Position/Profession
   1. Histotechnician/Histoscientist/Laboratory technician
   2. Pathologist
   3. Pathology resident
   4. Other
6. Did you attend the 'Manual IHC for Histotechnicians' virtual lecture series in January 2022?
   1. Yes
   2. No
   3. I do not know
7. Are you participating in the 'IHC for Histotechnicians & Pathologists Digital Mentorship Platform'?
   1. Yes
   2. No
   3. I do not know
8. Does your institution currently perform IHC, either manual or automated?
   1. Yes
   2. No
   3. I do not know
9. Since January 2022, have there been any positive changes in your laboratory facility in terms of space, staff, and resources for IHC capacity? (By positive, we mean changes needed to implement/improve/scale up IHC.)

|  | 1 - No changes at all. | 2 - A few changes. | 3 - Several changes. | 4 - Many changes. | 5 - Significant changes. | I do not know | We do no perform IHC |
| --- | --- | --- | --- | --- | --- | --- | --- |
| Laboratory space/equipment |  |  |  |  |  |  |  |
| Laboratory staff task reassignment |  |  |  |  |  |  |  |
| Resources for IHC (reagents, specimen containers, formalin, education materials) |  |  |  |  |  |  |  |
| Protocols/Standard Operating Procedures (SOPs) |  |  |  |  |  |  |  |
| Training |  |  |  |  |  |  |  |

Please list any examples, even if minor: _____________________________

1. If you or others at your institution are discussing plans to build IHC capacity, please select all items that are being discussed and indicate their level of priority.

|  | 1 – Not a priority at all | 2 – Slight priority | 3 – Somewhat of a priority | 4 – Very high priority | 5 – Highest priority | I do not know | We are not discussing plans to build IHC capacity |
| --- | --- | --- | --- | --- | --- | --- | --- |
| Implement IHC protocols |  |  |  |  |  |  |  |
| Train staff |  |  |  |  |  |  |  |
| Create or improve laboratory space |  |  |  |  |  |  |  |
| Obtain equipment, reagents, or other resources |  |  |  |  |  |  |  |
| Seek advice from experts or other institutions |  |  |  |  |  |  |  |

Please list any examples, even if minor: _____________________________

1. Since participating in the webinar, or participating in the digital mentorship platform, to what extent has your institution made positive changes to its IHC practices? (By positive, we mean changes needed to implement/improve/scale up IHC.)
   1. 1 - Not at all
   2. 2 - Slightly
   3. 3 - Somewhat
   4. 4 - A moderate extent
   5. 5 - Significantly
   6. I did not participate in the webinar, or participate in the digital mentorship platform
   7. We do not perform IHC.
   8. Please list any examples, even if minor: _____________________________
2. Since participating in the webinar, or participating in the digital mentorship platform, have you noticed improved quality of slides produced in your laboratory. (By quality we mean sensitivity and specificity so that the pathologist can make an accurate diagnosis).
   1. 1 - Not at all
   2. 2 - Slightly
   3. 3 - Somewhat
   4. 4 - A moderate extent
   5. 5 - Significantly
   6. I did not participate in the webinar, or participate in the digital mentorship platform
   7. We do not perform IHC.
   8. Please list any examples, even if minor: _____________________________
3. Since participating in the webinar, or participating in the digital mentorship platform, how often have you sought external opinions or assessments of the quality of slides in your laboratory?
   1. Never
   2. Rarely
   3. Sometimes
   4. Often
   5. Very often
   6. I did not participate in the webinar, or participate in the digital mentorship platform
   7. We do not perform IHC.
   8. Please list any examples, even if minor: _____________________________
4. Since participating in the webinar, or participating in the digital mentorship platform, how often have you used the lecture slides, lecture videos, or other webinar materials to train others?
   1. Never
   2. Rarely
   3. Sometimes
   4. Often
   5. Very often
   6. I did not participate in the webinar, or participate in the digital mentorship platform
   7. Please list any examples, even if minor: _____________________________
5. Did your institution begin implementing IHC after the "Manual IHC for Histotechnicians" webinar? If yes, how did your institution fund the start-up costs? Select all that apply.
   1. Donation from a private donor.
   2. My institution funded the implementation.
   3. Stakeholder investment/Investment by other organizations.
   4. Private funds/investment.
   5. We were awarded a grant.
   6. We have started implementing IHC but I do not know how it was funded.
   7. We have not started implementing IHC.
6. If your institution does NOT currently perform IHC, please describe your comfort level to implement manual IHC in your setup.
   1. Not comfortable at all
   2. Slightly comfortable
   3. Somewhat comfortable
   4. Very comfortable
   5. Extremely comfortable
   6. N/A (This question does not apply to me since we already perform IHC.)
7. I am interested in more information about access to IHC reagents. (Select all that apply)
   1. I would like to obtain a list of distributors to compare prices
   2. I would like to know how to be assured of the quality of reagents
   3. I would like to understand the process of arranging delivery of reagents through customs
   4. I would like to understand how to purchase, budget, and prioritize reagent and equipment purchases
   5. I would like to know how to plan for scaling up when case volume increases
8. Please describe any additional information you are seeking. _____________________________
9. Please freely share any perceived barriers to implementing IHC changes in your setup. _____________________________
10. Please freely share (1) any additional training needs (2) the best way for you to access training. _____________________________

**D.2 Section for institutions that do NOT currently perform IHC**

1. If your institution does NOT currently perform IHC, please describe your comfort level to implement manual IHC in your setup.
   1. 1 - Not comfortable at all
   2. 2 - Slightly comfortable
   3. 3 - Somewhat comfortable
   4. 4 - Very comfortable
   5. 5 - Extremely comfortable
   6. N/A (This question does not apply to me since we already perform IHC)
2. If your institution does NOT currently perform IHC, have you taken any steps to implement manual IHC in your setup? Select all that apply.
   1. We have discussed starting manual IHC with supervisors or leadership
   2. We have obtained or shared protocols
   3. We have conducted a needs assessment of space, staff, and required resources
   4. We have asked colleagues for advice on how to get started
   5. We have sought advice within our wider professional networks
   6. N/A (This question does not apply to me since we already perform IHC)

**E. 1-year Survey (Follow-up)**

1. Name
2. Email Address
3. Institution and Department
4. Country
5. Position/Profession
   1. Histotechnician/Histoscientist/Laboratory technician
   2. Pathologist
   3. Pathology resident
   4. Other
6. What is the main challenge you are currently facing in your pathology lab? _____________________________
7. Since January 2022, have there been any positive changes in your laboratory facility in terms of space, staff, and resources for IHC capacity? (By positive, we mean changes needed to implement/improve/scale up IHC.)

|  | 1 - No changes at all. | 2 - A few changes. | 3 - Several changes. | 4 - Many changes. | 5 - Significant changes. | I do not know | We do no perform IHC |
| --- | --- | --- | --- | --- | --- | --- | --- |
| Laboratory space/equipment |  |  |  |  |  |  |  |
| Laboratory staff task reassignment |  |  |  |  |  |  |  |
| Resources for IHC (reagents, specimen containers, formalin, education materials) |  |  |  |  |  |  |  |
| Protocols/Standard Operating Procedures (SOPs) |  |  |  |  |  |  |  |
| Training |  |  |  |  |  |  |  |

Please list any examples, even if minor: _____________________________

1. Since participating in the webinar, or participating in the digital mentorship platform, have you noticed improved quality of slides produced in your laboratory. (By quality we mean sensitivity and specificity so that the pathologist can make an accurate diagnosis).
   1. 1 - Not at all
   2. 2 - Slightly
   3. 3 - Somewhat
   4. 4 - A moderate extent
   5. 5 - Significantly
   6. I did not participate in the webinar, or participate in the digital mentorship platform
   7. We do not perform IHC.
   8. Please list any examples, even if minor: _____________________________
2. What are your/your institution’s IHC goals for 2023?
3. Which educational IHC topics would you like to learn about in2023? (Please provide specific details.)
